# Risk Factors for Postoperative Wound Infections in Patients with Brain Tumors Without Anticoagulant or Antiplatelet Therapy: A Ten-Year Single-Center Retrospective Analysis

**DOI:** 10.3390/jcm15030977

**Published:** 2026-01-26

**Authors:** Anatoli Pinchuk, Nikolay Tonchev, Anna Schaufler, Claudia A. Dumitru, Belal Neyazi, Klaus-Peter Stein, Ibrahim Erol Sandalcioglu, Ali Rashidi

**Affiliations:** Department of Neurosurgery, Otto-von-Guericke-University, 39120 Magdeburg, Germany; anatoli.pinchuk@med.ovgu.de (A.P.); nikolay.tonchev@med.ovgu.de (N.T.); anna.schaufler@med.ovgu.de (A.S.); claudia.dumitru@med.ovgu.de (C.A.D.); belal.neyazi@med.ovgu.de (B.N.); klaus-peter.stein@med.ovgu.de (K.-P.S.); erol.sandalcioglu@med.ovgu.de (I.E.S.)

**Keywords:** brain tumors, postoperative complications, wound infection, risk factors

## Abstract

**Background/Objectives**: This study aimed to identify risk factors for postoperative wound infections and healing disorders in patients with brain tumors, based on a large, single-center analysis, and to establish an evidence-based foundation for prevention. **Methods**: A retrospective analysis was conducted on 1480 patients who underwent intracranial tumor resection in our department over a ten-year period, without the influence of anticoagulant or antiplatelet medication. Potential predictors of wound healing disorders were evaluated, focusing on demographic variables and pre-existing conditions. **Results**: Among the 1480 patients, postoperative wound infections occurred in 47 cases, corresponding to a cumulative incidence of 3.17%. Platelet count (*p* = 0.018) and partial thromboplastin time (*p* = 0.011) emerged as potential risk factors for postoperative wound infections. Length of hospital stay appeared as a distinct outcome-associated marker in cases of postoperative wound infection (*p* = 0.018). In contrast, demographic characteristics (age, sex, blood type), comorbidities (hypertension, diabetes mellitus, cardiovascular disease, kidney disease, chronic inflammatory conditions), and other surgical or laboratory parameters showed no significant association with wound healing disorders. **Conclusions**: In patients with brain tumors undergoing surgery without the influence of anticoagulant or antiplatelet therapy, most demographic factors, common comorbidities, and selected laboratory parameters were not associated with an increased risk of postoperative wound infections. Awareness of the identified risk factors may help guide preventive strategies and nursing care.

## 1. Introduction

Postoperative wound infections remain a significant complication following neurosurgical procedures, with surgical site infections (SSIs) after craniotomy representing a particularly serious concern due to their proximity to the central nervous system [1].

These infections substantially increase patient morbidity, mortality, length of hospital stay, and healthcare costs while potentially compromising essential adjuvant oncological treatments including radiation therapy and chemotherapy [1,2], that can lead to permanent disability, prolonged hospitalization, and substantially increased healthcare costs [3]. Reported incidence rates for postoperative wound infection after craniotomy vary in the literature, ranging from 1.2% to 5.3% [3,4,5,6,7,8]. Craniotomy is one of the most frequently performed neurosurgical procedures and is widely used to treat intracranial pathologies, including brain tumors. The risk of infection in this setting is elevated due to several inherent factors: the involvement of critical neural structures, the technical complexity of the surgery, and the typically extended operative duration [9,10].

Given this information, clinical staff implement meticulous protocols to minimize the effect of such complications on patient outcomes. Multiple hospital infection control protocols are standard practice in healthcare and form part of everyday routine. The use of single-use devices, regular staff training, and monitoring of infection rates have become an integral part of modern surgical practice. Many hospitals nowadays are equipped with negative air pressure operating theatres, which, together with personal protective equipment, provide greater protection against droplet and airborne infections [11,12]

Multiple studies have identified potential contributors to the development of postoperative wound infections in craniotomy patients. These include prolonged operative time, diabetes mellitus, perioperative antibiotic use, and the specific surgical approach [10,13,14,15,16]. Such infections can have a substantial negative impact on patient outcomes. They may compromise the success of the primary treatment, increase healthcare expenditures, prolong hospital stays, raise the likelihood of readmission and reoperation, and in severe cases, pose a life-threatening risk [17]. Early identification of modifiable and non-modifiable risk factors is therefore critical for effective prevention and timely intervention.

Relatively few studies have examined the risk factors for wound infections specifically in patients undergoing craniotomy for brain tumors. Most prior research has focused on craniotomy in general and has identified risk factors such as advanced age, diabetes mellitus, malnutrition, preoperative steroid therapy, preoperative radiation, obesity, prolonged operative duration, clean-contaminated or contaminated surgical fields, and a history of recent neurosurgical procedures [18]. However, the findings across these studies have been inconsistent, and the specific subset of patients with brain tumors has not been adequately addressed. However, antiplatelet medication can increase the risk of postoperative wound complications, which can lead to infections at the surgical site. In addition, there is a risk of post-operative hemorrhage, which in turn increases the risk of wound infection [19,20].

Given these gaps, we conducted a large, single-center retrospective analysis of patients with brain tumors undergoing craniotomy, specifically excluding the influence of anticoagulant and antiplatelet therapy. The aim of this study was to evaluate potential demographic, clinical, surgical, and laboratory predictors of postoperative wound infections in this population, with the ultimate goal of providing a robust, evidence-based foundation for prevention and, when necessary, early treatment of this complication. These results reflect our experience of infection control procedures in neuro-oncological surgery over a period of one decade.

## 2. Materials and Methods

This retrospective, single-center study was conducted in the Department of Neurosurgery at university of Magdeburg, encompassing a ten-year period from 2008 to 2018.

A total of 1480 consecutive adult patients who underwent elective craniotomy for intracranial tumor resection were included. Patients receiving perioperative anticoagulants or antiplatelet therapy were excluded to eliminate the potential influence of these medications on postoperative wound healing. Additional exclusion criteria included pre-existing cranial wound infection, and incomplete clinical data.

Data was obtained from electronic medical records and operative reports. Collected variables included demographic characteristics (age, sex, blood type, smoking history), pre-existing comorbidities (hypertension, diabetes mellitus, cardiovascular disease, chronic kidney disease, chronic inflammatory conditions, chronic liver disease), and preoperative laboratory values (platelet count, partial thromboplastin time [PTT], prothrombin time, C-reactive protein, white blood cell count). Surgical parameters such as duration of surgery, surgical approach, and intraoperative findings were also recorded.

The primary outcome was the occurrence of postoperative wound infection or wound healing disorder within 30 days of surgery. Wound infection was defined according to the Centers for Disease Control and Prevention (CDC) criteria (heat, pain, redness, swelling, and functional limitations), including clinical signs of infection, microbiological confirmation, and/or the requirement for surgical revision or targeted antibiotic therapy.

### Statistical Analysis

Descriptive statistics were used to summarize baseline characteristics. Categorical variables were analyzed using the chi-square test or Fisher’s exact test, and continuous variables were compared using Student’s *t*-test or Mann–Whitney U test, depending on data distribution. Variables with *p* < 0.05 in univariate analysis were entered into a multivariate logistic regression model to identify risk factors for postoperative wound infections. Statistical significance was set at *p* < 0.05. Analyses were performed using R version 4.5.1 incorporating the packages gtsummary version 2.3.0 for statistical testing and ggplot2 version 3.5.2 as well as gghalves version 0.1.4 for visualization.

## 3. Results

During the study period, 1480 patients underwent intracranial tumor resection without the influence of perioperative anticoagulant or antiplatelet therapy. Postoperative wound infections occurred in 47 patients, corresponding to an overall incidence of 3.17%.

**Figure 1:**
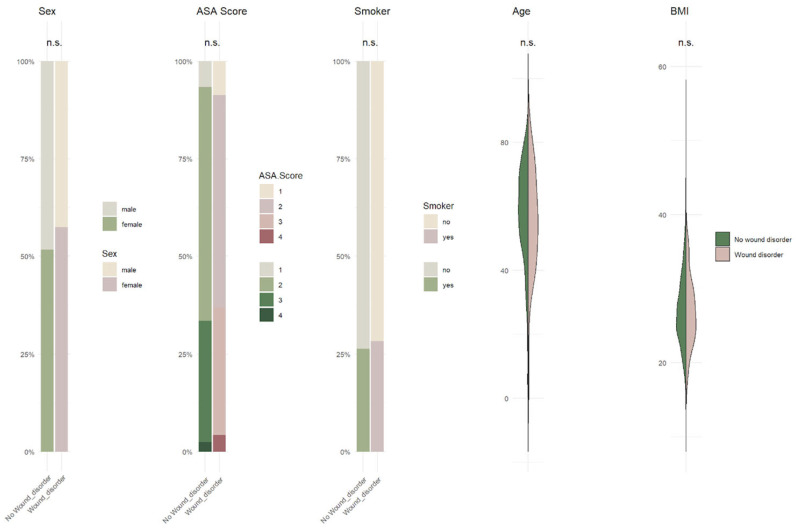
Graphic distribution of the influence of various demographic parameters on wound healing after cranial surgery. None of the listed parameters showed statistically significant in the univariate analyze.

The mean age of patients with wound healing disorders was 55.3 years, with no statistically significant difference compared with patients without such disorders (*p* = 0.337). Gender distribution was also not significantly associated with infection (*p* = 0.441); of the 47 affected patients, 27 (57.45%) were female. Other demographic factors, including body mass index (BMI) (*p* = 0.511) and smoking status (*p* = 0.773), showed no significant association with wound healing disorders (Table 1; Figure 1).

**Figure 2:**
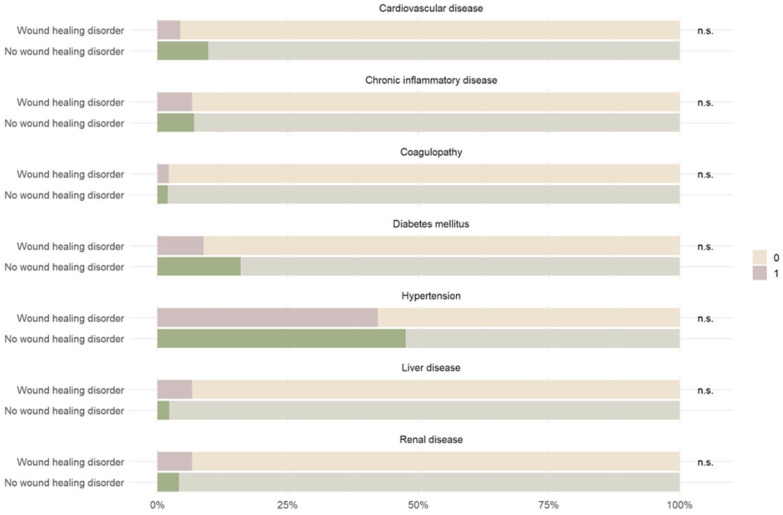
Graphic distribution of the influence of various comorbidities on wound healing after cranial surgery. Inferential statistics with a significance threshold of *p*-value ≤ 0.05.

The American Society of Anesthesiologists (ASA) classification did not differ significantly between groups (*p* = 0.758). Among 698 patients with arterial hypertension, 20 developed a postoperative wound infection (*p* = 0.184). Of 228 patients with diabetes mellitus, 4 developed an infection (*p* = 0.391), and among 142 patients with coronary heart disease, 2 developed an infection (*p* = 0.207). Neither chronic inflammation (*p* = 0.848) nor liver disease (*p* = 0.065) demonstrated a statistically significant association (Table 2; Figure 2).

**Figure 3:**
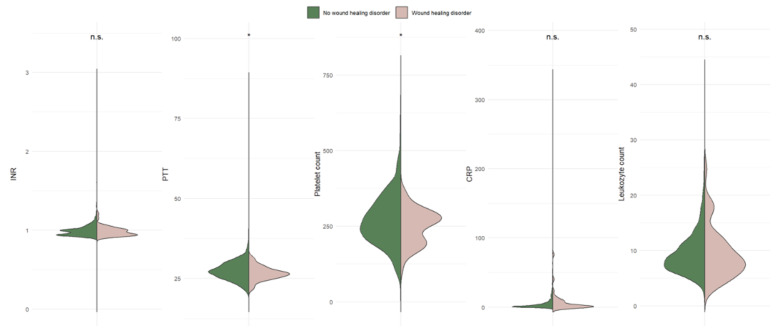
Graphic distribution of the influence of various laboratory parameters on wound healing subsequent to cranial surgery. These plot presentations employ the ggplot2 package to illustrate the descriptive statistical results regarding several preoperative laboratory parameters. The * shows the extreme outliers.

Laboratory analyses revealed no significant association between elevated C-reactive protein (CRP) levels and wound infection (*p* = 0.270) (Table 3; Figure 3). However, partial thromboplastin time (*p* = 0.011) and platelet count (*p* = 0.018) were significantly associated with postoperative wound infection. Other hematologic parameters, such as leukocyte count, showed no significant correlation (*p* = 0.679).

**Figure 4:**
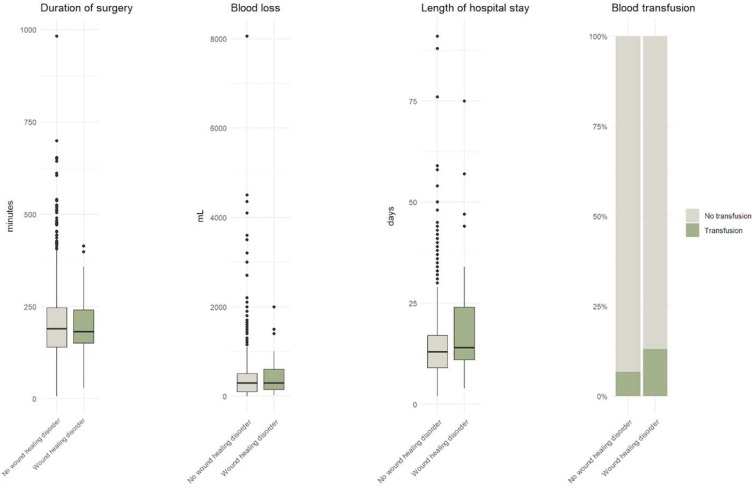
Graphic distribution of the influence of various surgical procedure related parameters on wound healing subsequent to cranial surgery in box plot technique. These charts provide a visual representation of the distribution of common values and variability for each separate parameter.

In patients with postoperative wound infection, the mean operative duration was 192.1 min (range 182.4–201.7 min), with no statistically significant difference compared to the non-infection group (*p* = 0.712). Intraoperative blood loss, although clinically relevant to wound healing, did not reach statistical significance (*p* = 0.251). Length of hospital stay was significantly longer in patients with postoperative wound infection (*p* = 0.018), which appeared as a strong outcome associated marker (Table 4; Figure 4). Table 5 provides an overview of the multivariate analysis of the parameters identified as significant.

**Table 4:** Surgical parameters that potentially influence wound healing following cranial surgery. Relevant results are marked in Bold.

		Wound Healing Disorder/Infection		
		No (N 1433) Mean ± SD	Yes (N 47) Mean ± SD	*p*-Value
**Surgical** **parameters**	Duration of the operation [min]	187.1 (175.6–198.6)	192.1 (182.5–201.7)	0.712
	Blood loss [mL]	299.6 (205.9–393.3)	359.5 (270.2–448.7)	0.251
	Duration of hospital stay (day)	13.6 (12.7–14.4)	17.5 (15.6–19.3)	**0.018**

**Table 5:** Presents the relevant parameters in multivariate analysis. The rest of the parameters were non-significant.

	OR	95%; CI	*p*-Value
PTT [s]	0.9	0.81; 1.00	0.060
Duration of stay [7 days]	1.35	1.15; 1.57	0.001
Platelet count [10 × 10/L]	0.97	0.94; 1.01	0.13

## 4. Discussion

Postoperative wound infections remain one of the most serious complications following craniotomy, with the potential to cause poor prognosis and even death [21]. Early diagnosis is often challenging, as the cerebrospinal fluid culture positivity rate is typically low and clinical signs may be nonspecific [22]. For these reasons, early identification of high-risk patients and the implementation of targeted preventive measures are crucial to reduce the incidence of wound infections and improve postoperative quality of life.

Several comorbidities have been reported to predispose surgical patients to wound infections. Diabetes mellitus and hypertension are among the most common underlying conditions in patients undergoing major surgery, and previous studies have demonstrated that preoperative diabetes significantly increases susceptibility to postoperative wound infections [23]. The impaired tissue repair and regeneration associated with diabetes, combined with a hyperglycemic environment that facilitates bacterial growth, are thought to contribute to this increased risk [23,24]. In contrast to these findings, our study did not identify diabetes mellitus or hypertension as significant risk factors for wound infection following intracranial tumor resection without Aspirin influence. This discrepancy may be attributable to strict perioperative glycemic control in our patient cohort, as well as the relatively small number of infection cases. In our postoperative care practice, day-to-day blood sugar level measurements are routinely interpreted. If necessary, timely correction of blood sugar levels is performed.

Advanced age has also been implicated as a risk factor in previous studies, with older patients showing increased wound infection rates due to reduced immune function, the presence of comorbidities, and decreased physiological reserves [25,26]. However, in our cohort, age was not significantly associated with postoperative wound infection, even when considering patients aged 60 years or older.

Obesity, often measured by BMI, has been linked to an elevated risk of surgical site infections, with potential mechanisms including reduced tissue perfusion in adipose tissue and nutritional deficiencies affecting immune competence [27,28]. Our results, however, did not demonstrate a significant association between BMI and postoperative wound infection, suggesting that BMI alone may be an insufficient predictor in this patient population.

Surgical factors are also important determinants of postoperative wound infection risk. Prolonged operative time has been consistently reported as a strong predictor [29,30], as longer surgeries increase the likelihood of bacterial contamination through multiple mechanisms, including extended exposure of the surgical site to airborne pathogens and repeated handling of tissues and instruments [3]. While operative duration was not a significant factor in our study, length of hospital stay emerged as a strong outcome-associated marker. This may reflect both the influence of postoperative complications on prolonged hospitalization and the potential for nosocomial exposure over time. Optimizing perioperative management and implementing strategies to reduce unnecessary hospitalization may therefore help lower infection risk. A prolonged hospital stay is often the result of poor patient recovery, immobilization and a decrease in the general immune protection mechanisms. Therefore, the length of hospital stay can be interpreted as an outcome-associated marker, as well as a risk factor for surgical site infections [11,12].

Other factors frequently cited in the literature, such as previous radiotherapy, incision type, tumor recurrence, and other comorbidities [31,32], were not significantly associated with wound infection in our analysis.

There is a relative paucity of studies focusing specifically on risk factors for wound infections following elective craniotomy for brain tumor resection. Our results contribute to filling this gap and provide neurosurgeons with data that may help identify high-risk groups and guide preventive strategies. Moreover, it raises a scientific discussion on the role of platelets as immunomodulatory cells, which have functions that extend far beyond hemostasis. Platelets secrete substances (PDGF, EGF, TGF-Beta as well as PF4) and express membrane proteins that form part of the immune defense environment to effectively remove pathogens [33]. Therefore, a low platelet count poses a risk not only for hemorrhagic complication due to impaired hemostasis, but also for infectious complications. As a part of the wound healing cascade, platelets play a central role in the clot formation phase and, later during the proliferation phase, in angiogenesis. However, our current analysis did not specifically study these possible correlations.

Furthermore, it would be important to research the possible cellular and molecular pathways. These include, for example, changes in oxygen supply associated with age and hormones, stress, medications, substance abuse, and nutrition.

Butyrylcholinesterase (BChE), a nonspecific cholinesterase enzyme, has been associated with the risk of progression of liver dysfunction and, more recently, with infectious diseases and septic shock, with ongoing research investigating the utility of BChE in various systemic inflammatory conditions [34,35]. Furthermore, contemporary data from retrospective observational studies indicate that low BChE levels are independent predictors of severe systemic inflammation, with this phenomenon occurring early in the inflammatory cascade. This finding raises the possibility of minimizing time delays between clinical assessment and treatment of the underlying inflammatory drivers, such as surgical site infection [34,35,36].

A better understanding of these influencing factors may lead to therapies that improve wound healing and eliminate damaged wounds. Further prospective, multicenter studies are warranted to validate these findings and to refine risk stratification models for this patient population.

## 5. Conclusions

In this large, single-center retrospective analysis of patients undergoing elective craniotomy for brain tumor resection without the influence of anticoagulant or antiplatelet therapy, postoperative wound infections occurred in 3.17% of cases. Platelet count and partial thromboplastin time were identified as significant factors associated with wound infection, whereas demographic characteristics, comorbidities, and most laboratory and surgical parameters showed no significant association. These findings suggest that, beyond established demographic and comorbidity profiles, specific perioperative laboratory values and postoperative course indicators may be more relevant for predicting infection risk in this patient group.

Our results highlight the importance of routine perioperative monitoring of coagulation parameters, particularly platelet count and partial thromboplastin time, as part of a targeted infection prevention strategy in neurosurgical patients. Early identification and correction of abnormalities in these parameters may reduce postoperative infection risk. Furthermore, strategies aimed at minimizing hospital stay, such as enhanced recovery protocols and optimized perioperative care, could contribute to lowering the incidence of wound infections. By focusing preventive measures on these modifiable factors, neurosurgical teams may improve patient outcomes and reduce healthcare costs.

In summary, this study showed that certain laboratory parameters, such as platelet count and partial thromboplastin time, were risk factors for postoperative wound infection in patients with brain tumors. On the other hand, predictors such as demographic data (age, gender, blood type) and pre-existing conditions (high blood pressure, diabetes, cardiovascular disease, kidney disease, chronic inflammation) show no significant evidence of a connection with wound healing disorders. The results can serve as a reference for clinical medical staff to strengthen the assessment and management of patients undergoing brain tumor surgery and to take targeted and effective measures as early as possible to prevent the occurrence of postoperative wound infection in patients with brain tumors and improve clinical outcomes. A possible limitation of our research is the lack of description and comparison of infection control procedures, which play an additional role as unmeasurable confounding factor in preventing SSIs. At the same time, due to the limited number of articles included in this study, some risk factors may have a particular influence on the research results. Future large-sample, multicenter, high-quality research is recommended.

## Figures and Tables

**Table 1:** Demographic parameters that potentially influence wound healing following cranial surgery.

			Wound Healing Disorder/Infection		
			No N 1433 (%), Mean ± SD	Yes N 47 (%), Mean ± SD	*p*-Value
**Demographic parameters**	Sex	Female Male	742 (51.74) 692 (48.26)	27 (57.45) 20 (42.55)	0.441
	Age		57.68 ± 15.78	55.30 ± 16.60	0.337
	BMI		27.36 ± 4.46	26.92 ± 4.46	0.511
	ASA-Classification	I II III–IV	95 (6.65) 855 (59.87) 478 (33.47)	4 (8.7) 25 (54.35) 17 (36.96)	0.758
	Smoker	Yes No	370 (26.35) 1034 (71.74)	13 (28.26) 33 (71.74)	0.773

**Table 2:** Patients’ comorbidities that potentially influence wound healing following cranial surgery.

			Wound Healing Disorder/Infection		
			No N 1433 (%)	Yes N 47 (%)	*p*-Value
**Comorbidity**	Hypertension	Yes No	678 (47.28) 756 (52.72)	20 (42.55) 27 (57.45)	0.184
	Diabetes Type 1/2	Yes No	224 (15.62) 1210 (84.38)	4 (8.51) 43 (91.49)	0.391
	Liver disease	Yes No	32 (2.23) 1402 (97.77)	3 (6.38) 44 (93.62)	0.065
	Cardiovascular disease	Yes No	140 (9.76) 1294 (90.24)	2 (4.26) 45 (95.74)	0.207
	Chronic inflammation	Yes No	102 (7.11) 1332 (92.89)	3 (6.38) 44 (93.62)	0.848

**Table 3:** Laboratory that potentially influence wound healing following cranial surgery. Relevant results are marked in Bold.

		Wound Healing Disorder/Infection		
		No (N 1433) Mean ± SD	Yes (N 47) Mean ± SD	*p*-Value
**Laboratory parameters**	INR	1.00 (0.95–1.06)	0.983 (0.982–0.984)	0.139
	PTT [s]	27.44 (±3.57)	26.59 (±2.12)	**0.011**
	Platelet count [10 × 9/L]	269.49 (±86.95)	248.85 (±55.65)	**0.018**
	C-reactive protein [mg/L]	4.6 (0.4–8.9)	3.5 (0.71–6.3)	0.270
	Leukocytes [Gpt/L]	9.2 (8.8–9.5)	8.9 (8.4–9.4)	0.679

## Data Availability

The datasets obtained and analyzed during the current study are available from the corresponding author on reasonable request.

## Abbreviations

The following abbreviations are used in this manuscript:

ASA	American Society of Anesthesiologists
BMI	Body mass index
CDC	Centers for Disease Control and Prevention
CRP	C-reactive protein
PTT	partial thromboplastin time
SSI	surgical site infection
